# Characteristics and Health Risks of Trace Metals in PM_2.5_ Before and During the Heating Period over Three Years in Shijiazhuang, China

**DOI:** 10.3390/toxics13040291

**Published:** 2025-04-10

**Authors:** Qingxia Ma, Shuangshuang Zou, Dongli Hou, Qingxian An, Peng Wang, Yunfei Wu, Renjian Zhang, Jinting Huang, Jing Xue, Lei Gu

**Affiliations:** 1 College of Geographical Science, Faculty of Geographical Science and Engineering, Henan University, Zhengzhou 450046, China; mqx@henu.edu.cn; 2 Key Laboratory of Geospatial Technology for the Middle and Lower Yellow River Regions (Henan University), Ministry of Education, Kaifeng 475004, China; zcszhr@henu.edu.cn; 3 Henan Key Laboratory of Integrated Air Pollution Control and Ecological Security, Kaifeng 475004, China; 4 Hebei Province Ecology Environmental Monitoring Center, Shijiazhuang 050000, China; hdlhbemc@163.com (D.H.); hbanqingxian@163.com (Q.A.); wptcyj@163.com (P.W.); 5 Key Laboratory of Middle Atmosphere and Global Environment Observation (LAGEO), Institute of Atmospheric Physics, Chinese Academy of Sciences, Beijing 100029, China; wuyf@mail.iap.ac.cn (Y.W.); zrj@tea.ac.cn (R.Z.); 6 College of Surveying and Mapping Engineering, Yellow River Conservancy Technical Institute, Kaifeng 475004, China; jenkins1204@126.com; 7 School of Ecology and Environment, Northwestern Polytechnical University, Xi’an 710129, China; xuejing0089@nwpu.edu.cn

**Keywords:** haze pollution, heating, human health, North China Plain

## Abstract

To explore the characteristics of PM_2.5_ and assess the health risks to residents in Shijiazhuang before and during the heating period in 2019, 2020 and 2021, the hourly concentrations of PM_2.5_ and its nine selected trace elements were determined. The results showed that the mass concentrations of PM_2.5_ were 80.32 ± 50.21 μg m^−3^ (2019), 69.97 ± 41.91 μg m^−3^ (2020) and 58.70 ± 41.97 μg m^−3^ (2021) during the heating period, representing greatly improved air quality. The PM_2.5_ levels in the heating period were 1.04~1.60 times greater than those before the heating period, while the total selected trace element concentrations were about 1.44~1.97 times higher, indicating that strict control for PM_2.5_ in the heating period should be imposed. The overall hazard quotient (HQ) of the nine selected trace elements in the heating period were 1.08~1.42 times higher than those before the heating period, while the total cancer risks (CR) were decreased by 29.04% (2020) and 3.50% (2021). There were high health risks not only in local areas, but also in the south of Hebei, the north of Henan, and southern and central Shanxi. The health risks increased by 1.21~2.26 times from clean levels to heavy pollution levels. The leading element of HQ was Mn, while the dominant elements of CR varied from As to Co. Increases in PM_2.5_ concentrations and HQ from before the heating period to during the heating period were observed, and there was even an inverse CR change between before the heating period and during the heating period, further identifying that air pollution control was efficient.

## 1. Introduction

Fine particulate matter (PM_2.5_) is widely acknowledged as a key particulate pollutant in the air, and causes adverse health impacts [1,2,3]. As critical components of PM_2.5_, trace elements have caused serious health outcomes and public health challenges [4,5]. Trace elements, including As, Mn, Cr, Zn, Co, Ni, and Cu in ambient PM_2.5_ undergo multi-pathway exposure (inhalation, ingestion, and dermal permeation), and then exhibit bioaccumulative potential in adipose tissue and parenchymal organs, finally inducing various diseases [6,7,8]. Therefore, an investigation and assessment of the health risks of PM_2.5_-associated trace elements are necessary [9,10].

Risk assessment models are applied to the estimation of potential health risks in relation to exposure to trace elements, assessing both non-cancer and cancer risk effects [11]. Some researchers have commonly focused on the trace element concentrations and assessed their high speciation-dependent health risks [12,13,14]. Gao et al. [15] identified that high-risk thresholds for both non-cancer and cancer trace elements during the haze events were prevalent. Liu et al. [16] noted that Mn was the main factor contributing to the total non-cancer risks, and Cr had greater cancer risks in four mid-sized cities in key urban agglomerations. Li et al. [17] measured the concentration and fractionation of PM_2.5_-associated trace elements in Hebei and concluded that the most important contributors to both non-cancer and cancer risks were industrial processes and soil dust during clean days, while coal combustion dominated during moderate to severe haze episodes. However, the health risks of separate elements in PM_2.5_ were assessed using concentration levels and spatial distribution data. Seasonal variations categorized into the non-heating period and heating period have not been clearly determined.

Shijiazhuang, situated in the Beijing–Tianjin–Hebei region (BTH) of China and serving as the capital of Hebei Province, ranks among the most air-polluted cities in the world [18], especially during the heating period [19,20]. The economic development of Shijiazhuang is mainly based on industry, such as pharmaceuticals, textiles, and mining, with coal as the main source of energy [21]. Liang et al. [12] reported that significant disparities between the heating and non-heating periods were attributed to increasing coal combustion and deteriorating meteorological conditions. In addition, some observations across the BTH region have documented a significant elevation in urban PM_2.5_ concentrations from the non-heating period to the heating period [22].

Therefore, in this study, hourly PM_2.5_ concentrations and nine trace elements of PM_2.5_ before and during the heating period in Shijiazhuang from 2019 to 2021 were measured with the aims of (1) characterizing the PM_2.5_-bound trace element concentrations in consecutive years and pollution episodes; (2) comparing variations in PM_2.5_ concentration and apportioned non-cancer and cancer risks of trace elements of PM_2.5_ in different periods and years using a health risk assessment method; and (3) identifying the potential areas of high health risk of trace elements via a potential source contribution function (PSCF). This quantification is good for identifying the most harmful trace elements impacting human health, and providing a scientific basis for enhancing pollution control strategies in other highly polluted regions of China.

## 2. Materials and Methods

### 2.1. Observational Site

**Figure 1:**
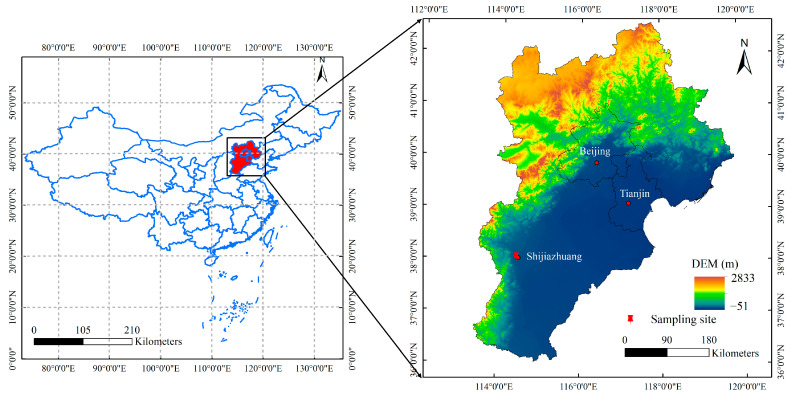
Location of observational site in Shijiazhuang.

Shijiazhuang, serving as the capital of Hebei Province, spans 2240 km^2^ and is consistently ranked among the world’s most polluted cities. Sampling activity was conducted to collect PM_2.5_ at Hebei University of Economics and Business in Hebei, China (38.06° N, 114.45° E), which is located in a district with Zhangshi Highway 600 m to the northwest. The sampling site was set on the residential area and the specific location of the observational site is presented in Figure 1. According to the heating time in Shijiazhuang, the entire measurement period was divided into before the heating period (25 October–14 November from 2019 to 2021) and the heating period itself (15 November–3 December from 2019 to 2021) in this research.

### 2.2. Sample Collection and Laboratory Analysis

From 25 October 2019 to 3 December 2021, atmospheric PM_2.5_ concentration quantification was conducted through a four-channel ambient air sampler (XHPM2000E, Hebei Xianhe Instrument Ltd., Shijiazhuang, China) with a time interval of 1 h. An aerosol monitoring station was established on the rooftops of buildings, with an approximate elevation of 15 m over ground level. Both field blanks and parallel samples were obtained at the same time before and during the heating period over three years for the purpose of quality assurance/quality control (QA/QC). Elements in PM_2.5_ were acquired through online measurements (XHAM-2000A, Hebei Xianhe Instrument Ltd., China). The MDLs (1 h resolution, ng m^−3^) during the measurement are shown in Table S1.

### 2.3. Analysis Methods

#### 2.3.1. Health Risk Assessment Model

The selected trace elements in PM_2.5_ that pose non-cancer and cancer risks to humans were considered using the inhalation exposure pathway in this study. The trace element exposure dose rate of non-cancer and cancer was calculated by computing the lifetime average daily dose (LADD_i_) for inhalation, and evaluated using Equation (1):

(1) $$\mathrm{ADD}\left(\mathrm{LA}{\mathrm{DD}}_{i}\right)=\frac{c_{i}\;\times \;\mathrm{InhR}\;\times \;\mathrm{EF}\;\times \;\mathrm{ED}}{\mathrm{AT}\;\times \;\mathrm{BW}}$$

with c_i_ representing the trace element i concentration (mg/m^3^). The parameters relevant to the evaluation of health risks are displayed in Table S2. HQ_i_ is the hazard quotient, which represents the non-cancer risk of trace element i, and was calculated using Equation (2):

(2) $$HQ_{i}=\mathrm{LAD}D_{i}/\mathrm{RfDinh}$$

(3) $$\mathrm{HI}=\sum {\mathrm{HQ}}_{i}$$

RfD_inh_ is a receivable risk dose (mg/kg/day) for metals (Table S3). The hazard index (HI) was computed to evaluate the non-cancer risk impacts. The calculation formula was computed using Equation (4):

(4) $$R_{i}=\mathrm{LAD}D_{i}\times \mathrm{SFI}$$

(5) $$R_{t}=\sum R_{i}$$

The inhalation risk of the trace element i (R_i_) is computed; the SFI refers to slope factors of inhalation (kgday/mg, Table S3). The bearable risk value for R_i_ ranges from 10^−6^ to 10^−4^ [23,24,25]; an R_i_ value more than 10^−4^ indicates a serious cancer risk; an R_i_ value less than 10^−6^ shows that the risk is acceptable [26].

#### 2.3.2. Potential Source Contribution Function

Potential health risk regions in Shijiazhuang before and during the heating period from 2019 to 2021 were quantified in this study. The parcel trajectories affecting Shijiazhuang were computed in 1 h intervals for 24 h, and the air masses of the dominating pollutant before and during the heating period were identified. The study area was separated into ij grid cells in the PSCF, and the ij grid cells were 0.5° × 0.5° in this study. The PSCF value was defined as following formula:

(6) $${\mathrm{PSCF}}_{\mathrm{ij}}=\frac{m_{\mathrm{ij}}}{n_{\mathrm{ij}}}$$

where n_ij_ is the number of endpoints that are distributed in the ij grid cell, and m_ij_ stands for the endpoint number that exceeds the limit. W(n_ij_) is the weighting function to decrease uncertainty caused by tiny values of n_ij_, and n_ave_ represents each grid average number in the PSCF. The function W_ij_ is defined as:

(7) $${\mathrm{WPSCF}}_{\mathrm{ij}}=\frac{m_{\mathrm{ij}}}{n_{\mathrm{ij}}}W(n_{\mathrm{ij}})$$

(8) $$W(n_{\mathrm{ij})}=\left\{\begin{array}{c} 1.0,n_{\mathrm{ij}}>3\;n_{\mathrm{ave}}\\ 0.7,1.5\;n_{\mathrm{ave}}<n_{\mathrm{ij}}\leq {3\;n}_{\mathrm{ave}}\\ 0.4,n_{\mathrm{ave}}<n_{\mathrm{ij}}\leq 1.5\;n_{\mathrm{ave}}\\ 0.2,n_{\mathrm{ij}}\leq n_{\mathrm{ave}} \end{array}\right.$$

## 3. Results and Discussion

### 3.1. PM_2.5_ Variations and Its Trace Element Concentrations Before and During the Heating Period

The concentrations of PM_2.5_ in the heating period in Shijiazhuang were 80.32 ± 50.21 μg m^−3^ (2019), 69.97 ± 41.91 μg m^−3^ (2020) and 58.70 ± 41.97 μg m^−3^ (2021), respectively, which were 1.60 (2019), 1.04 (2020), and 1.07 (2021) times higher than those before the heating period, respectively. Compared to that during the heating period of 2019, the concentration of PM_2.5_ reduced by 12.89% (2020) and 26.92% (2021). The continuous decline in PM_2.5_ concentrations in the heating period from 2019 to 2021 was likely due to the Blue Sky Defense Battle in 2018 [27], which mitigates severe air pollution and safeguards public health.

As can be seen in Table 1, the metal element concentrations during the heating period were higher than those before the heating period. The total metal element concentrations ranged from 382.44 to 778.21 ng m^−3^ during the heating period over three years, which was 1.44~1.97 times higher than that before the heating period. Concentrations of both PM_2.5_ and nine selected metal elements showed an increasing trend during the heating period, indicating that control of PM_2.5_ in the heating period should be imposed. The mean mass concentration of Zn was consistently the highest from 2019 to 2021, up to 547.37 ± 414.49 ng m^−3^, and V was the lowest, ranging from 0.11 to 1.05 ng m^−3^ in three years. The concentration of As was 2.79 ng m^−3^ during the heating period in 2019, representing a slight decrease in 2020 with a concentration of 2.69 ng m^−3^, but an obvious increase in 2021 with a concentration of 5.79 ng m^−3^. The concentrations of Mn during the heating period in 2020 and 2021 were significantly higher than the heating period in 2019. The concentration of Co was 12.51 ng m^−3^ during the heating period in 2019, showing a dramatic decrease with a concentration of 2.94 ng m^−3^ (2020) and 2.93 ng m^−3^ (2021). In particular, one of the most interesting observations was that the concentration of Cu in the heating period in 2020 was evidently about 2.96~5.93 times greater than other heating periods. The higher concentration of Cu in winter was also found in Beijing (200.01 ng m^−3^), Baoding (190.21 ng m^−3^) [27] and Linfen (120.10 ng m^−3^) [28].

In this study, the average concentrations of Cr, Zn, Pb, Ni had increases during the heating period than before the heating period over three years. During the heating period, Cr was 1.87 (2019), 1.03 (2020) and 1.51 (2021) times higher than before the heating period. Compared to before the heating period, Zn increased by 63.55% (2019), 82.54% (2020) and 73.86% (2021) during the heating period. The mass concentrations of Pb and Ni in the heating period were 1.32~1.56 times and 1.39~1.91 times higher, respectively, than those observed in before the heating period over three years. Thus, the levels of Cr, Zn, Pb, Ni during the heating period should be carefully controlled because of the increases in metal concentration. Zn and Pb originate from vehicle exhaust [29,30,31,32], and debris from worn tires and brake pads [32,33,34]. Industrial emissions are associated with the steel and metallurgical industries, with high levels of Cr and Ni [35,36]. Hence, the control of vehicle emissions and industrial emissions in the heating period should be considered as priorities.

### 3.2. Health Risk Assessment Before and During the Heating Period

**Figure 2:**
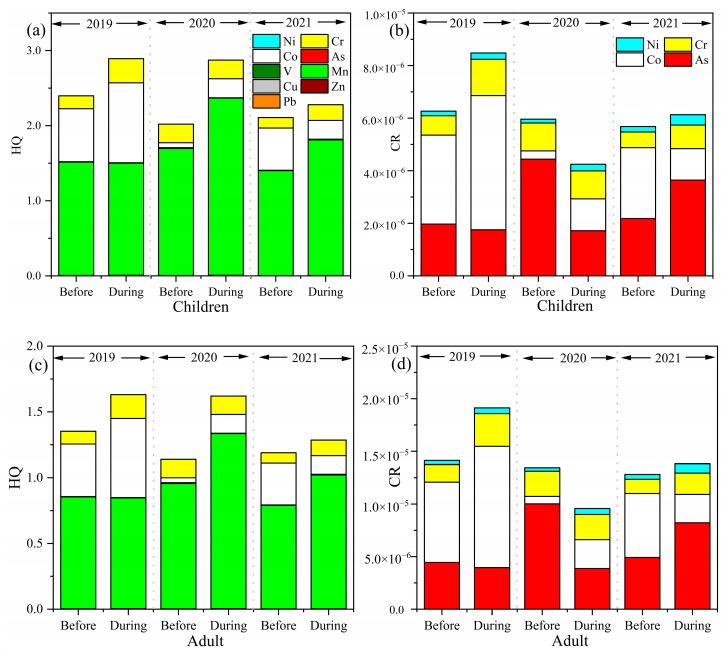
Health risks related to non-cancer (HQ) and cancer (CR) trace elements before and during the heating period over three years. HQ (**a**) and CR (**b**) for children, HQ (**c**) and CR (**d**) for adults.

The non-carcinogenic and carcinogenic risks of the selected trace elements for adults and children before and during the heating period are displayed in Figure 2 and Tables S7–S9. For adults, the average HQ values of nine trace elements in 2019 were 1.35 (before the heating period) and 1.63 (during the heating period), respectively. Compared to before the heating period in 2019, the average HQ values of the nine trace elements decreased by 15.56% (2020) and 11.85% (2021), and the mean HQ values dropped by 1.84% (2020) and 20.86% (2021) compared to the heating period in 2019. The results indicated a non-cancer risk although the average HQ values showed a declining trend over three years. The CR values achieved 1.91 × 10^−5^ during the heating period in 2019, which was 1.35 times higher than before the heating period. The CR of the selected trace elements during the heating period were 9.58 × 10^−6^ (2020) and 1.38 × 10^−5^ (2021), which were 29.04% (2020) and 3.50% (2021) lower than before the heating period.

The HQ and CR in three years before and during the heating period for adults were all above the acceptable limit, which showed that there was both a non-carcinogenic and a carcinogenic risk in Shijiazhuang. It should be noted that the HQ of Mn was highest in the heating period over three years, accounting for 51.62% (2019), 83.18% (2020) and 79.19% (2021) of the overall HQ. Mn predominantly originated from manufacturing associated with steel and metallurgical smelting [20,37]. Previous research found coal combustion [38] and vehicle exhaust [39,40] with high explained variation of Mn, indicating that the control measures could not efficiently reduce either domestic or traffic-related coal consumption. The HQ value of Mn was also reported to be highest in Qingdao and Shijiazhuang by Liu et al. [1] and Wang et al. [41]. During the heating period, the CR of Ni were 1.39 (2019), 1.74 (2020) and 1.91 (2021) times higher than before the heating period over three years, indicating that Ni should be controlled. The CR value of Co (accounting for 60.22%) was the highest during the heating period in 2019, while As was in 2020 (accounting for 40.42%) and 2021 (accounting for 59.41%), which was consistent with the total CR.

For children, the mean HQ values of the nine trace elements in 2019 were 2.40 (before the heating period) and 2.89 (during the heating period), respectively (Figure 2). The change rates of average HQ values before and during the heating period for children were similar to adults in 2020 and 2021, and the HQ exceeded the acceptability limit (1.00 × 10^−6^). The HQ values of Mn (1.49, 2.33 and 1.81) in the heating period over three years were greater than other trace elements, followed by Pb (6.75 × 10^−3^, 6.20 × 10^−3^ and 4.87 × 10^−3^) and As (4.50 × 10^−3^, 4.35 × 10^−3^ and 9.35 × 10^−3^). The CR values of the nine trace elements over three years in the heating period were 8.48 × 10^−6^, 4.25 × 10^−6^ and 6.13 × 10^−6^, respectively, which were exceeded the acceptable limit (1 × 10^−6^), showing a carcinogenic risk. The CR values of As (1.75 × 10^−6^, 1.69 × 10^−6^ and 3.64 × 10^−6^) in the heating period were the highest, followed by that of Co (5.11 × 10^−6^, 1.21 × 10^−6^ and 1.20 × 10^−6^) and Cr (1.38 × 10^−6^, 1.06 × 10^−6^ and 8.99 × 10^−7^), while that of Ni was the lowest (2.41 × 10^−7^, 2.49 × 10^−7^ and 3.94 × 10^−7^). As was a crucial element driving carcinogenic risk and should be given more attention in Shijiazhuang. This is consistent with the research by Diao [42]. High concentrations of As mainly originated from coal combustion [43,44,45]. Wang et al. [28] found that As was the key element contributing to the cancer risk in Linfen, while Cd and Ni were carcinogenic to humans in Changzhi [46], and As, Cr, Ni and Co in Huangshi [47].

The non-cancer risk for children during the heating period was 1.8 times greater than for adults, while the carcinogenic risk for adults was 2.3 times greater than for children. This phenomenon is similar to the results of prior risk assessments carried out in other major Chinese cities [48]. Adults had a higher carcinogenic risk, which has been linked to the fact that the potential duration of exposure for adults was longer than that of children [33]. The non-carcinogenic and carcinogenic risks were mainly attributed to Mn and As during the heating period, respectively. This finding was similar to that of Behera et al. [49] and Lin et al. [50]. In addition, the HQ and CR of the nine selected trace elements were significantly decreased during the heating period, indicating that the air pollution control strategies are effective.

### 3.3. Potential Health Risk Regions Before and During the Heating Period

**Figure 3:**
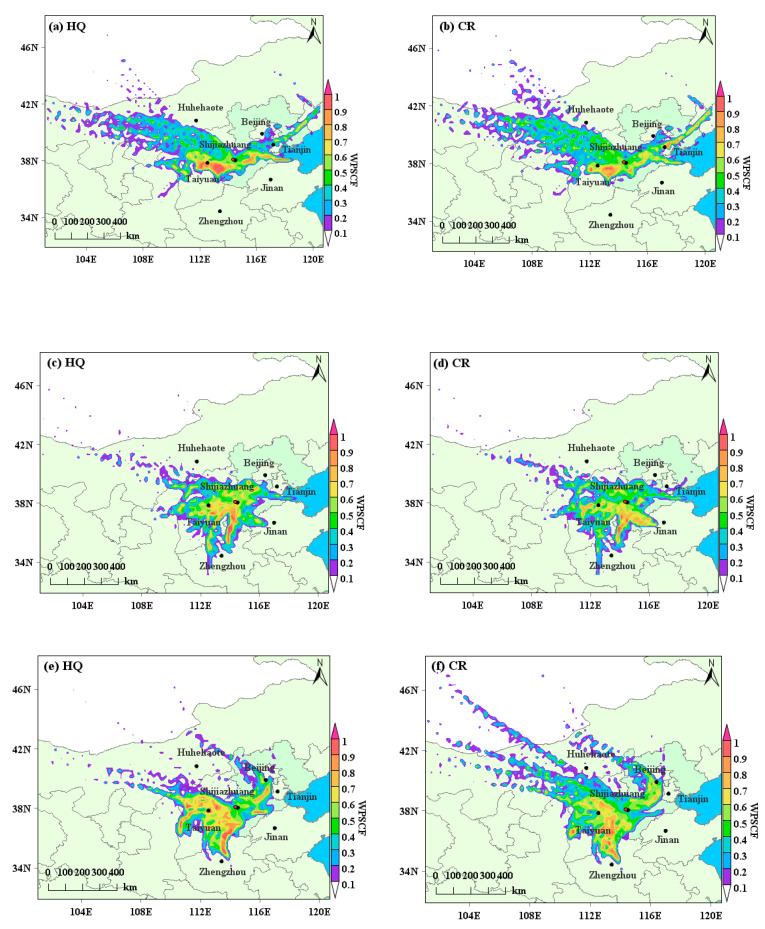

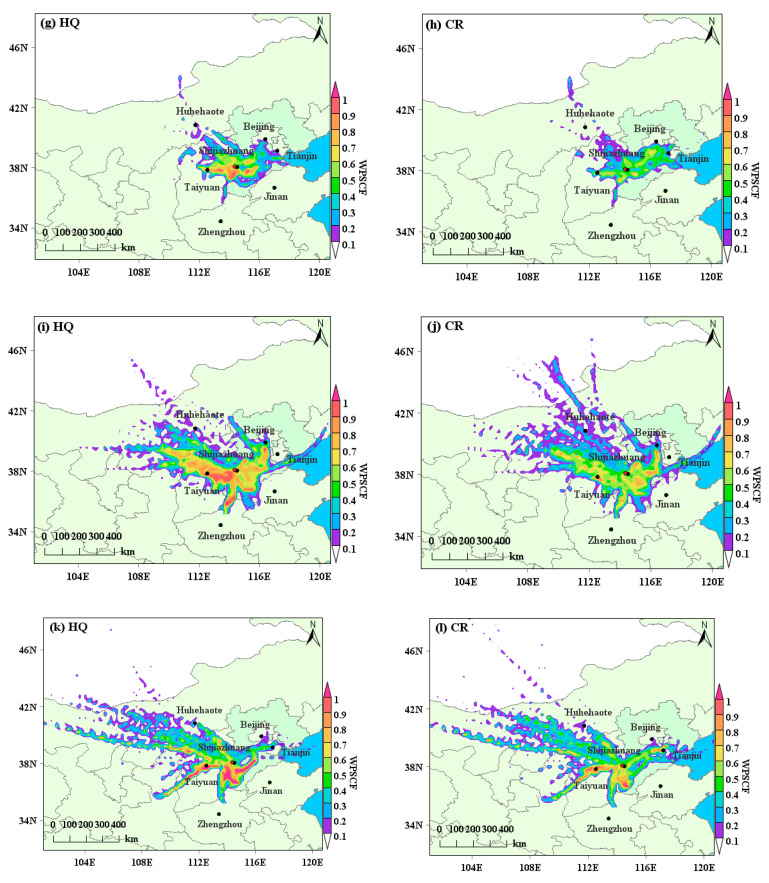
The WPSCF for health risks of selected trace elements before and during the heating period over three years, (**a**–**d**) were in 2019, (**e**–**h**) in 2020, and (**i**–**l**) in 2021. WPSCF for HQ (**a**,**e**,**i**) and CR (**b**,**f**,**j**) before the heating period, and HQ (**c**,**g**,**k**) and CR (**d**,**h**,**l**) in the heating period for three years, respectively.

PSCF methods were applied to assess the public health impacts of regional sources from various PM_2.5_-associated sources in air on local human health. The potential non-cancer and cancer risk areas affecting the health of adults and children in Shijiazhuang were consistent. Before the heating period in 2019 (Figure 3a,b), the potential areas of high non-cancer risk were mainly located in eastern Shanxi, covering Taiyuan and Jinzhong; and eastern to southern Hebei, covering Cangzhou and Hengshui. The potential high cancer risk regions were mainly in east Shanxi, including Jinzhong; east to north of Tianjin; and Hebei, covering Tangshan. During the heating period in 2019 (Figure 3c,d), there were higher non-cancer risk values in the south of Hebei, covering Xingtai and Handan; the north of Henan, including Anyang and Hebi; and the east of Shanxi, covering Jinzhong. The potential high cancer risk regions were in the south of Hebei, covering Xingtai and Handan; northern Henan, including Anyang; and Shandong Liaocheng.

Before the heating period in 2020 (Figure 3e,f), the potential regions for high non-cancer risks and cancer risks were located in central Shanxi Province, covering Xinzhou, Taiyuan and Jinzhong; south of Beijing; north to west of Henan, including Anyang and Hebi; and south of Hebei, covering Xingtai and Handan. For the heating period in 2020 (Figure 3g,h), the potential high non-cancer and cancer risks areas were located in the south to west of Hebei, covering Xingtai, and in the east of Shanxi, covering Jinzhong and Yangquan. Before the heating period in 2021, high health risks were primarily caused by the air masses transported from central and southern Hebei, covering Hengshui, Xingtai and Handan; and from central and eastern Shanxi, covering Shuozhou, Xinzhou, Jinzhong and Yangquan (Figure 3i,j). During the heating period in 2021 (Figure 3k,l), there were higher health risks in the south to west of Hebei, covering Hengshui, Xingtai and Handan; and in the central and southwest of Shanxi, covering Taiyuan and Linfen.

Generally, the high risk areas affecting adults and children’s health in Shijiazhuang could be southern and south-eastern Hebei, central and south-eastern Shanxi, and northern and north-western Henan, which is similar to the previous study in Shijiazhuang [42].

### 3.4. Variations in Health Risks During Different Pollution Levels Before and During the Heating Period

#### 3.4.1. Concentrations of Trace Elements as Pollution Worsens

**Figure 4:**
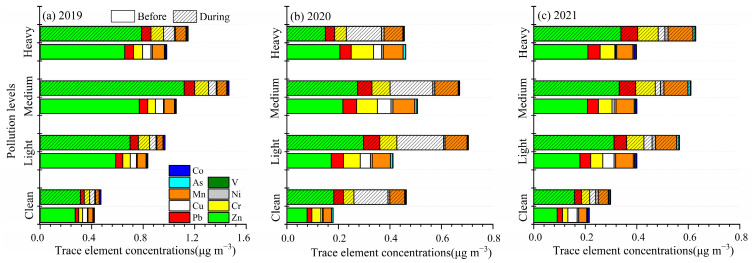
The trace element concentrations in PM_2.5_ at different pollution levels before and during the heating period over three years.

In recent years, the haze pollution during the heating period in Beijing-Tianjin-Hebei region (BTH) occurred more frequently and seriously than during non-heating periods [51]. Therefore, to identify the concentration characteristics and the health risks of the nine trace elements in different air pollution levels, four typical pollution levels were identified, including 0 to 75 μg m^−3^ (clean), 75 to 115 μg m^−3^ (light pollution), 115 to 150 μg m^−3^ (medium pollution) and over 150 μg m^−3^ (heavy pollution). Figure 4 shows the concentrations of the nine selected trace elements at different levels over three years. As PM_2.5_ pollution increased, the concentrations of the individual elements increased, while their total contribution to PM_2.5_ (percentage) tended to decrease with increasing pollution levels. This result was consistent with that of Cheng et al. [38]

Before the heating period in 2019, the concentrations of selected trace elements were mostly lowest during the clean level. As PM_2.5_ pollution increased, the concentrations of Zn, Pb, As, Mn, Cr and Cu clearly increased. The concentrations of Zn and Pb increased by 2.16 and 2.01 (light), 2.83 and 2.42 (medium), and 2.41 and 2.49 (heavy) times, respectively. The concentrations of As and Mn increased by 1.95 and 1.79 (light), 2.43 and 2.10 (medium), and 2.57 and 2.46 (heavy) times, respectively. The concentrations of Cr and Cu increased by 1.92~2.34 and 1.29~1.74 times, respectively. During the heating period in 2019, the concentrations of selected trace elements increased from low to high levels of pollution. The Zn concentration at the medium level was clearly higher than other pollution levels, and was 3.58 times higher than at the clean level. Compared with the clean level, the concentrations of As and Pb increased by 2.47 and 2.14 (light), 4.67 and 2.71 (medium), and 3.37 and 2.44 (heavy) times during the heating period, respectively. The concentrations of Cr, Mn and Cu increased by 2.09~2.60, 1.71~2.89 and 1.26~2.14 times during the heating period, respectively.

Before the heating period in 2020, the total concentrations of nine trace elements were 2.20 (light), 2.77 (medium) and 2.63 (heavy) times greater than at the clean level. For example, the concentrations of Cu, Zn and Mn increased by 6.03, 2.20 and 2.18 (light), 8.09, 2.77 and 2.60 (medium), and 4.80, 2.63 and 2.38 (heavy) times, respectively. The concentrations of Pb, Cr and As increased by 2.37~2.88, 1.87~2.48 and 1.41~1.64 times, respectively. Additionally, the concentration of Co at medium pollution was 11.07 times greater than at the clean level. During the heating period in 2020, the total concentration of nine selected elements in light and medium pollution were 1.51 and 1.64 times greater than at the clean level, while the heavy pollution level concentration was similar to the clean level. The concentrations of Cr, Mn and Cu increased by 11~72% in light pollution, 27~59% in medium pollution and 3~37% in heavy pollution compared to the clean level, respectively.

Before the heating period in 2021, in contrast to the clean level, the concentrations of Zn and Mn increased by 1.97 and 2.19 (light), 2.29 and 2.27 (medium), and 2.31 and 2.02 (heavy) times, respectively. The concentration of Pb increased by 2.03~2.30 times than at the clean level. At the clean and light pollution levels, the concentrations of Cu were 0.03 and 0.04 μg m^−3^. As pollution levels increased, the Cu concentration increased to 0.08 μg m^−3^. During the heating period in 2021, compared with the clean level, the concentrations of Mn, Pb and Cr increased by 2.10, 1.81 and 2.12 (light), 2.40, 2.41 and 2.42 (medium), and 2.49, 2.42 and 2.52 (heavy) times during the heating period, respectively. The concentrations of Zn and As increased by 1.97~2.13 and 1.73~2.02 times compared to the clean level, respectively.

As the pollution levels increased, the element concentrations in PM_2.5_ demonstrated an increasing trend, especially for the concentrations of Zn, Cu, Cr, Pb and Mn at the medium pollution level in 2019 and the light and medium pollution levels in 2020 during the heating period. This phenomenon can be attributed to bad atmospheric diffusion conditions (high RH and low wind speed) (Figure S1).

#### 3.4.2. Health Risks of Trace Elements at Different Pollution Levels

**Figure 5:**
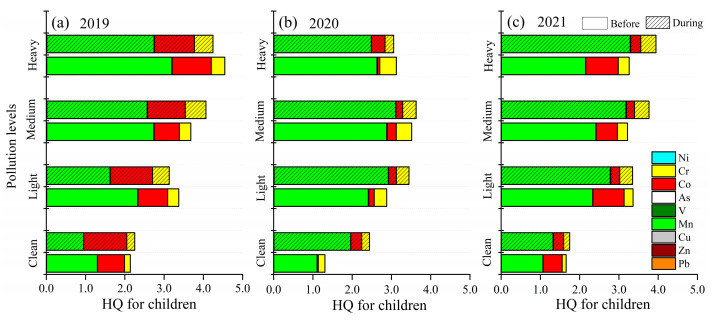

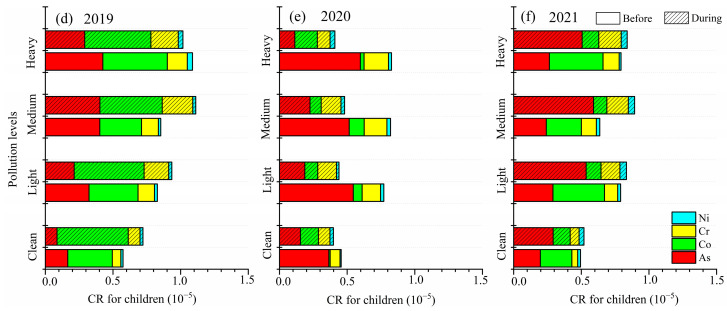
The trace elements in HQ and CR for children at different pollution levels before and during the heating period over three years.

**Figure 6:**
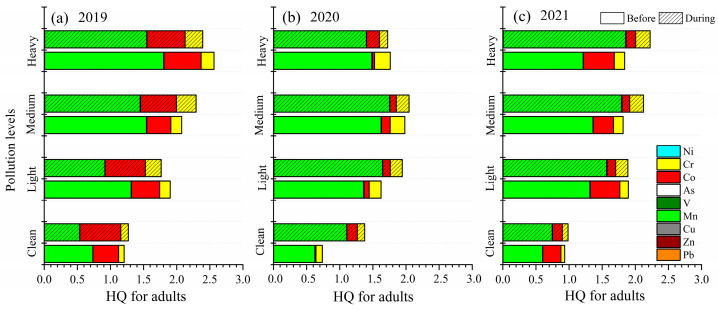

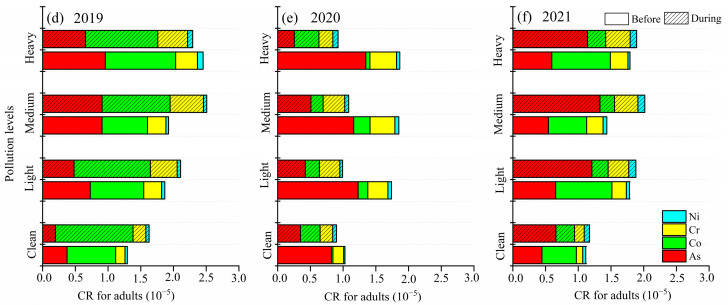
The trace elements in HQ and CR for adults at different pollution levels before and during the heating period over three years.

Before the heating period in 2019, the total HQ value of the nine selected trace elements increased as the pollution level increased (Figure 5 and Figure 6). Mn generally contributed to the HQ, and the average proportions of Mn were 60.75% (clean), 68.84% (light), 73.91% (medium) and 69.89% (heavy). During the heating period in 2019, the total HQ values of Co were 1.10 and 1.08 at the clean and medium pollution levels. As the pollution level worsened, the total CR value of the nine selected trace elements showed an obvious increasing trend before the heating period in 2019. The CR value of As (3.73 × 10^−6^ for adults and 1.66 × 10^−6^ for children) was 1.95 (light), 2.43 (medium) and 2.57 (heavy) times higher than at the clean level. The CR value of Cr (1.42 × 10^−6^ for adults and 6.31 × 10^−7^ for children) was 1.92 (light), 1.97 (medium) and 2.34 (heavy) times higher than at the clean level. The CR value of Co and Ni increased by 0.94~1.45 and 1.09~2.35 times compared to the clean level, respectively. During the heating period in 2019, the CR of As for adults and children reached 1.95 × 10^−6^ and 8.63 × 10^−7^, respectively. With the pollution level increasing, the CR of As was 2.47 (light), 4.67 (medium) and 3.37 (heavy) times greater than that at the clean level. The CR of Cr was 2.09 (light), 2.60 (medium) and 2.33 (heavy) higher than at the clean level.

Before the heating period in 2020, the HQ values of Mn and Co were 2.18 and 6.55 (light), 2.60 and 11.07 (medium) and 2.38 and 2.76 (heavy) times higher than at the clean level, respectively (Figure 5 and Figure 6). The HQ value of Cr increased by 1.87~2.48 times compared to the clean level. In addition, the HQ of Cu at the clean level for adults and children reached 4.45 × 10^−5^ and 7.89 × 10^−5^, respectively, which was 6.03 (light), 8.09 (medium) and 4.80 (heavy) times higher. During the heating period in 2020, the total HQ values showed an obvious increasing trend at the light and medium pollution levels, which were 1.94 and 2.05 for adults and 3.45 and 3.63 for children, respectively. Before the heating period in 2020, As was the most carcinogenic metal, and the average proportions of As were 79.82% (clean), 70.69% (light), 62.73% (medium) and 72.19% (heavy). During the heating period in 2020, the CR of As (3.48 × 10^−6^ for adults and 1.54 × 10^−6^ for children) was 1.21 (light) and 1.46 (medium) times higher than at the clean level. The CR value of Cr and Ni increased by 1.11~1.72 times and 1.04~1.38 times compared to the clean level.

Before the heating period in 2021, the HQ value of the selected trace elements represented a clear increasing trend with pollution levels increasing (Tables S7–S12). The HQ values of Mn and Cr were 2.19 and 2.26 (light), 2.27 and 2.56 (medium) and 2.02 and 2.75 (heavy) times higher than at the clean level. During the heating period in 2021, the average proportions of Mn were 75.43% (clean), 82.69% (light), 84.08% (medium) and 83.25% (heavy) at different pollution levels. The HQ value of Cr was 2.12 (light), 2.41 (medium) and 2.52 (heavy) times higher than at the clean level. Before the heating period in 2021, the CR value of the selected trace elements represented a clear increasing trend. The CR values of Co and Ni increased by 1.11~1.70 times and 1.05~1.22 times compared to the clean level. During the heating period in 2021, the CR of As (1.97 × 10^−6^ for adults and 2.92 × 10^−6^ for children) increased by 1.84 (light), 2.02 (medium) and 1.73 (heavy) times higher than the clean level.

In sum, the HQ was mainly due to Mn, while the dominant elements of CR changed from As to Co from the clean level to the light, medium, and heavy pollution levels. Therefore, as pollution levels have increased, the elements of Co need to be considered because of more adverse impacts on human health.

## 4. Conclusions

The temporal variation and health risks of nine trace metals in PM_2.5_ were investigated from 22 October to 3 December in 2019, 2020 and 2021 in Shijiazhuang. The average concentration of the nine selected trace elements accounted for 0.97%, 0.77% and 0.65% of the PM_2.5_ mass during the heating period over three years, of which Zn was the highest. The PM_2.5_ concentrations and trace metal elements during the heating period were greater than before the heating period, further illustrating that the heating activity was positively related to the increased air pollution. Compared to the heating period in 2019, the PM_2.5_ concentrations dropped by 12.89% (2020) and 26.92% (2021) during the heating period, indicating that air pollution control was efficient in recent years. Additionally, the results found that the total HQ of the nine selected trace elements during the heating period was 1.63 for adults and 2.89 for children in 2019 using the health risk assessment model. The HQ of the nine selected trace elements were 1.60 (2020) and 1.29 (2021) for adults, and 2.87 (2020) and 2.28 (2021) for children during the heating period. The total CR were 1.91 × 10^−5^ for adults and 8.48 × 10^−6^ for children during the heating period in 2019, while the total CR were decreased to 9.58 × 10^−6^ (adults) and 4.25 × 10^−6^ (children) in 2020, 1.38 × 10^−5^ (adults) and 6.13 × 10^−6^ (children) in 2021. The health risks elevated as the pollution level increased and the dominant elements of CR were Co at the light, medium and heavy pollution levels. Hence, the control of Co should be strengthened, especially for critical sources during pollution episodes in Shijiazhuang. It seems to be a good way to eliminate the trace elements that are most harmful to human health.

## Figures and Tables

**Table 1:** Mean mass concentrations of 9 trace metals before and during the heating period from 2019 to 2021 in Shijiazhuang.

Species	2019		2020		2021	
	Before	During	Before	During	Before	During
PM_2.5_	50.15 ± 34.82	80.32 ± 50.21	67.50 ± 48.65	69.97 ± 41.91	54.74 ± 41.06	58.70 ± 41.97
Pb	32.69 ± 22.51	48.89 ± 29.70	28.75 ± 42.06	44.92 ± 29.09	26.72 ± 18.40	35.28 ± 21.60
Cu	40.06 ± 46.82	49.54 ±56.24	18.77 ± 48.79	146.75 ± 87.75	34.11 ± 60.47	24.73 ± 45.55
Mn	44.42 ± 26.29	43.95 ± 34.28	48.04 ± 47.05	68.51 ± 35.46	41.10 ± 35.05	53.13 ± 37.24
V	0.73 ± 4.63	0.00 ± 0.00	0.11 ± 0.93	0.58 ± 2.64	1.05 ± 3.42	0.86 ± 2.80
As	3.13 ± 3.35	2.79 ± 3.54	6.28 ± 6.54	2.69 ± 3.47	3.47 ± 3.84	5.79 ± 4.98
Co	8.29 ± 6.90	12.51 ± 9.57	0.74 ± 3.27	2.94 ± 4.02	6.61 ± 7.33	2.93 ± 4.76
Cr	35.46 ± 20.49	66.28 ± 38.27	49.11 ± 41.03	50.37 ± 34.74	28.69 ± 18.83	43.15 ± 26.49
Ni	4.95 ± 5.99	6.89 ± 7.78	3.99 ± 7.38	7.12 ± 8.68	5.90 ± 8.12	11.26 ± 10.05
Zn	334.67 ± 268.71	547.37 ± 414.49	118.63 ± 139.67	216.56 ± 160.10	118.09 ± 77.97	205.30 ± 122.93
Total	504.40 ± 405.68	778.21 ± 593.86	274.99 ± 336.78	540.45 ± 365.96	265.79 ± 237.08	382.44 ± 276.40

PM_2.5_ concentration in μg m^−3^, and trace metal concentration in ng m^−3^.

## Data Availability

The data presented in this study are available on request from the corresponding author.

## Supplementary Materials

The following supporting information can be downloaded at: https://www.mdpi.com/article/10.3390/toxics13040291/s1, Figure S1: The relationship between PM_2.5_ and wind direction and wind speed during different pollution levels in three years; Table S1: The minimum detection limits (MDLs) of elements in PM_2.5_; Table S2: Parameters used in the evaluation of non-carcinogenic and carcinogenic health risks; Table S3: The risk doses and inhalation slope factors (SFI) of trace elements; Table S4: Statistical analysis for meteorological parameters during the whole studying period; Table S5: Statistical analysis for meteorological parameters before and during the heating period; Table S6: Rating criteria and related information for Air Quality Index; Tables S7–S9: Health risks associated with cancer and non-cancer trace elements before and during the heating period in 2019, 2020 and 2021; Tables S10 and S11: Health risk related to cancer and non-cancer trace elements for different pollution levels before and during the heating period in 2019; Tables S12 and S13: Health risk related to cancer and non-cancer trace elements for different pollution levels before and during the heating period in 2020; Tables S14 and S15: Health risk related to cancer and non-cancer trace elements for different pollution levels before and during the heating period in 2021. References [52,53,54,55,56] are cited in the supplementary materials.

## Abbreviations

The following abbreviations are used in this manuscript:

HQ	Hazard Quotient
CR	Cancer Risk
