# Supplementary material for: Characteristics and Health Risks of Trace Metals in PM2.5 Before and During the Heating Period over Three Years in Shijiazhuang, China
Source: Toxics. 2025 Apr 10;13(4):291. doi: 10.3390/toxics13040291 (PMC12031353; doi:10.3390/toxics13040291)
Supplement: Supplementary file 1 [file toxics-13-00291-s001.zip › toxics-3554902-supplementary.pdf]

toxics MDPI

Characteristics and health risks of trace metals in $PM_{2.5}$ before and during heating periods of three years in Shijiazhuang, China

Qingxia Ma [1,2], Shuangshuang Zou [1,2], Dongli Hou [3], Qingxian An [3], Peng Wang [3], Yunfei Wu [4], Renjian Zhang [4], Jinting Huang [5], Jing Xue [6] and Lei Gu [1,2,*]

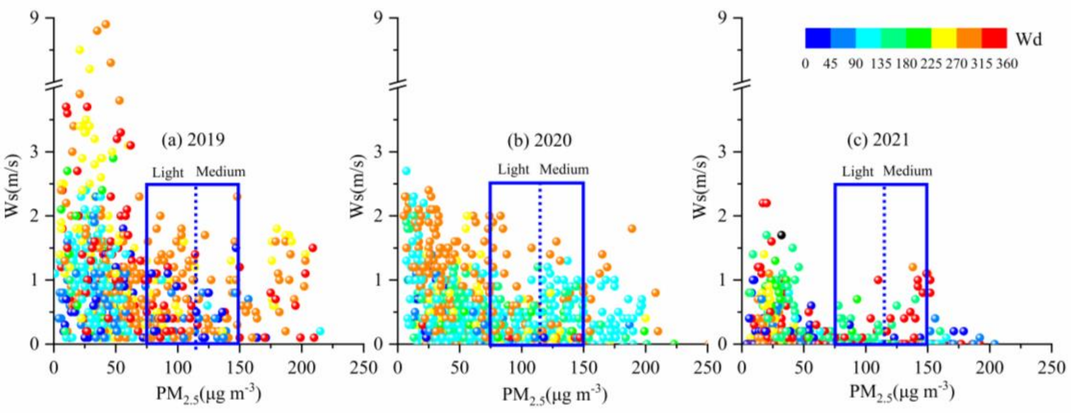

Figure S1. The relationship between $PM_{2.5}$ and wind direction and wind speed during different pollution levels in three years.

Table S1. The minimum detection limits (MDLs) of elements in $PM_{2.5}$

Elements	MDLs	Elements	MDLs	Elements	MDLs
K	1.51	Se	0.16	V	0.13
Ni	0.10	As	0.12	Ti	0.28
Si	14.9	Zn	0.29	Ca	0.33
S	5.8	Cu	0.33	Mn	0.10
Cl	3.2	Co	0.30	Fe	0.24
Br	0.23	Cr	0.12	Pb	0.17

Note: the concentration of elements is 1 h resolution and trcae metal concentration in ng m^{-3}.

Table S2. Parameters used in the evaluation of non-carcinogenic and carcinogenic health risks.

Parameters	Abbreviation	Children	Adult
Inhalation rate	InhR	7.6 m^3/d	20 m^3/d
Average body weight	BW	15 kg	70 kg
Exposure duration	ED	6 years	24 years
Exposure frequency	EF	350 d/year	350 d/year
Average time (non-carcinogens)	AT	ED × 365 days	ED × 365 days
Average time (carcinogens)	AT	70 × 365 days	70 × 365 days

[1]Parameters were obtained from US EPA. (2011) and Feng et al. (2016).

Table S3. The risk doses and inhalation slope factors (SFI) of trace elements.

Element	RfD_{inh}	SFI
Cr（VI）	1.00E-04	5.00E-01
Mn	1.43E-05	/
Co	5.71E-06	9.80E+00
Ni	2.06E-02	8.40E-01
Cu	4.02E-02	/
Zn	3.00E-01	/
As	3.01E-04	1.51E+01
Pb	3.52E-03	/
V	7.00E-03	/

[1]The reference values were acquired from Alves et al. (2019), Chen et al. (2014), and Wang et al. (2020).

Table S4. Statistical analysis for meteorological parameters in the whole studying period.

Types	T (℃)	RH (%)	WS (m/s)
2019	8.94	66.37	1.37
2020	10.01	59.79	0.52
2021	11.02	58.67	0.09

[1]T represents temperature, RH represents relative humidity, WS represents wind speed.

Table S5. Statistical analysis for meteorological parameters before and during heating periods.

Types	T (℃)		RH (%)		WS (m/s)	
	Before	During	Before	During	Before	During
2019	13.07	3.71	68.02	64.28	1.52	1.17
2020	13.81	5.20	48.24	74.41	0.51	0.53
2021	13.08	8.15	59.48	57.55	0.04	0.17

[1]T represents temperature, RH represents relative humidity, WS represents wind speed.

Table S6. Rating criteria and related information for Air Quality Index. Source: Technical Regulation on Ambient Air Quality Index (on trial) (HJ 633–2012) China.

AQI	Air Quality Situation	Air Quaality Level	$PM_{2.5}$ Concentration
0-50	Excellent	Class A	< 35
51-100	Good	Class B	35-75
101-150	Light pollution	Class C	75-115
151-200	Medium pollution	Class D	115-150
>200	Heavy pollution	Class E	>150

Table S7. Health risks associated with cancer and non-cancer trace elements before and during the heating period in 2019.

Period	Elements	Adults				Children			
		$ADD_{non-cancer}$	HQ	$LADD_{cancer}$	CR	$ADD_{non-cancer}$	HQ	$LADD_{cancer}$	CR
2019 Before heating	Cu	1.10E-05	2.73E-04			1.95E-05	4.84E-04		
	Mn	1.22E-05	8.51E-01			2.16E-05	1.51E+00		
	Pb	8.96E-06	2.54E-03			1.59E-05	4.51E-03		
	V	2.01E-07	2.87E-05			3.57E-07	5.10E-05		
	Zn	9.17E-05	3.06E-04			1.63E-04	5.42E-04		
	As	8.58E-07	2.85E-03	2.94E-07	4.44E-06	1.52E-06	5.06E-03	1.30E-07	1.97E-06
	Co	2.27E-06	3.98E-01	7.79E-07	7.63E-06	4.03E-06	7.06E-01	3.45E-07	3.38E-06
	Cr(VI)	9.72E-06	9.72E-02	3.33E-06	1.67E-06	1.72E-05	1.72E-01	1.48E-06	7.38E-07
	Ni	1.36E-06	6.58E-05	4.65E-07	3.90E-07	2.40E-06	1.17E-04	2.06E-07	1.73E-07
	Total		1.35E+00		1.41E-05		2.40E+00		6.27E-06
2019 During heating	Cu	1.36E-05	3.38E-04			2.41E-05	5.99E-04		
	Mn	1.20E-05	8.42E-01			2.14E-05	1.49E+00		
	Pb	1.34E-05	3.80E-03			2.38E-05	6.75E-03		
	V	0.00E+00	0.00E+00			0.00E+00	0.00E+00		
	Zn	1.50E-04	5.00E-04			2.66E-04	8.86E-04		
	As	7.64E-07	2.54E-03	2.62E-07	3.95E-06	1.35E-06	4.50E-03	1.16E-07	1.75E-06
	Co	3.43E-06	6.00E-01	1.18E-06	1.15E-05	6.08E-06	1.06E+00	5.21E-07	5.11E-06
	Cr(VI)	1.82E-05	1.82E-01	6.23E-06	3.11E-06	3.22E-05	3.22E-01	2.76E-06	1.38E-06
	Ni	1.89E-06	9.16E-05	6.47E-07	5.44E-07	3.35E-06	1.62E-04	2.87E-07	2.41E-07
	Total		1.63E+00		1.91E-05		2.89E+00		8.48E-06

Table S8. Health risks associated with cancer and non-cancer trace elements before and during the heating period in 2020.

Period	Elements	Adults				Children			
		$ADD_{non-cancer}$	HQ	$LADD_{cancer}$	CR	$ADD_{non-cancer}$	HQ	$LADD_{cancer}$	CR
2020 Before heating	Cu	5.14E-06	1.28E-04			9.12E-06	2.27E-04		
	Mn	1.32E-05	9.20E-01			2.33E-05	1.63E+00		
	Pb	7.88E-06	2.24E-03			1.40E-05	3.97E-03		
	V	3.00E-08	4.29E-06			5.33E-08	7.61E-06		
	Zn	3.25E-05	1.08E-04			5.76E-05	1.92E-04		
	As	1.87E-06	6.20E-03	6.40E-07	9.67E-06	3.31E-06	1.10E-02	2.84E-07	4.29E-06
	Co	2.04E-07	3.57E-02	6.99E-08	6.85E-07	3.62E-07	6.33E-02	3.10E-08	3.04E-07
	Cr(VI)	1.35E-05	1.35E-01	4.61E-06	2.31E-06	2.39E-05	2.39E-01	2.05E-06	1.02E-06
	Ni	1.09E-06	5.31E-05	3.75E-07	3.15E-07	1.94E-06	9.42E-05	1.66E-07	1.40E-07
	Total		1.14E+00		1.35E-05		2.02E+00		5.97E-06
2020 During heating	Cu	4.02E-05	1.00E-03			7.13E-05	1.77E-03		
	Mn	1.88E-05	1.31E+00			3.33E-05	2.33E+00		
	Pb	1.23E-05	3.50E-03			2.18E-05	6.20E-03		
	V	1.59E-07	2.28E-05			2.82E-07	4.04E-05		
	Zn	5.93E-05	1.98E-04			1.05E-04	3.51E-04		
	As	7.38E-07	2.45E-03	2.53E-07	3.82E-06	1.31E-06	4.35E-03	1.12E-07	1.69E-06
	Co	8.05E-07	1.41E-01	2.76E-07	2.70E-06	1.43E-06	2.50E-01	1.22E-07	1.20E-06

	Cr(VI)	1.38E-05	1.38E-01	4.73E-06	2.37E-06	2.45E-05	2.45E-01	2.10E-06	1.05E-06
	Ni	1.95E-06	9.47E-05	6.69E-07	5.62E-07	3.46E-06	1.68E-04	2.97E-07	2.49E-07
	Total		1.60E+00		9.58E-06		2.87E+00		4.25E-06

Table S9. Health risks associated with cancer and non-cancer trace elements before and during the heating period in 2021.

Period	Elements	Adults				Children			
		$ADD_{non\text{-}cancer}$	HQ	$LADD_{cancer}$	CR	$ADD_{non\text{-}cancer}$	HQ	$LADD_{cancer}$	CR
2021 Before heating	Cu	9.34E-06	2.32E-04			1.66E-05	4.12E-04		
	Mn	1.13E-05	7.87E-01			2.00E-05	1.40E+00		
	Pb	7.32E-06	2.08E-03			1.30E-05	3.69E-03		
	V	2.88E-07	4.11E-05			5.10E-07	7.28E-05		
	Zn	3.24E-05	1.08E-04			5.74E-05	1.91E-04		
	As	9.50E-07	3.16E-03	3.26E-07	4.92E-06	1.68E-06	5.60E-03	1.44E-07	2.18E-06
	Co	1.81E-06	3.17E-01	6.21E-07	6.08E-06	3.21E-06	5.62E-01	2.75E-07	2.70E-06
	Cr(VI)	7.86E-06	7.86E-02	2.70E-06	1.35E-06	1.39E-05	1.39E-01	1.19E-06	5.97E-07
	Ni	1.62E-06	7.84E-05	5.54E-07	4.65E-07	2.86E-06	1.39E-04	2.46E-07	2.06E-07
	Total		1.19E+00		1.43E-05		2.11E+00		6.32E-06
2021 During heating	Cu	6.77E-06	1.69E-04			1.20E-05	2.99E-04		
	Mn	1.46E-05	1.02E+00			2.58E-05	1.81E+00		
	Pb	9.67E-06	2.75E-03			1.71E-05	4.87E-03		
	V	2.36E-07	3.37E-05			4.18E-07	5.97E-05		
	Zn	5.62E-05	1.87E-04			9.97E-05	3.32E-04		
	As	1.59E-06	5.27E-03	5.44E-07	8.22E-06	2.81E-06	9.35E-03	2.41E-07	3.64E-06
	Co	8.03E-07	1.41E-01	2.75E-07	2.70E-06	1.42E-06	2.49E-01	1.22E-07	1.20E-06
	Cr(VI)	1.18E-05	1.18E-01	4.05E-06	2.03E-06	2.10E-05	2.10E-01	1.80E-06	8.99E-07
	Ni	3.09E-06	1.50E-04	1.06E-06	8.89E-07	5.47E-06	2.66E-04	4.69E-07	3.94E-07
	Total		1.29E+00		1.38E-05		2.28E+00		6.13E-06

Table S10. Health risk related to cancer and non-cancer trace elements for different pollution levels before the heating period in 2019.

Pollution levels before heating period	Elements	Adults				Children			
		$ADD_{non\text{-}cancer}$	HQ	$LADD_{cancer}$	CR	$ADD_{non\text{-}cancer}$	HQ	$LADD_{cancer}$	CR
Clean	Cu	1.02E-05	2.53E-04			1.81E-05	4.49E-04		
	Mn	1.05E-05	7.31E-01			1.85E-05	1.3E+00		
	Pb	7.45E-06	2.12E-03			1.32E-05	3.75E-03		
	V	2.44E-07	3.49E-05			4.33E-07	6.19E-05		
	Zn	7.45E-05	2.48E-04			1.32E-04	4.41E-04		
	As	7.21E-07	2.40E-03	2.47E-07	3.73E-06	1.28E-06	4.25E-03	1.10E-07	1.66E-06
	Co	2.21E-06	3.87E-01	7.58E-07	7.43E-06	3.92E-06	6.87E-01	3.36E-07	3.29E-06
	Cr(VI)	8.30E-06	8.30E-02	2.85E-06	1.42E-06	1.47E-05	1.47E-01	1.26E-06	6.31E-07
	Ni	1.27E-06	6.15E-05	4.34E-07	3.65E-07	2.25E-06	1.09E-04	1.93E-07	1.62E-07

	Total		1.21E+00		1.30E-05		2.14E+00		5.74E-06
Light pollution	Cu	1.31E-05	3.27E-04			2.33E-05	5.79E-04		
	Mn	1.87E-05	1.31E+00			3.31E-05	2.32E+0		
	Pb	1.50E-05	4.26E-03			2.66E-05	7.56E-03		
	V	0.00E+00	0.00E+00			0.00E+00	0.00E+00		
	Zn	1.61E-04	5.36E-04			2.85E-04	9.51E-04		
	As	1.41E-06	4.67E-03	4.82E-07	7.28E-06	2.49E-06	8.29E-03	2.14E-07	3.23E-06
	Co	2.43E-06	4.26E-01	8.34E-07	8.17E-06	4.31E-06	7.55E-01	3.70E-07	3.62E-06
	Cr(VI)	1.60E-05	1.60E-01	5.47E-06	2.73E-06	2.83E-05	2.83E-01	2.42E-06	1.21E-06
	Ni	1.69E-06	8.22E-05	5.80E-07	4.88E-07	3.00E-06	1.46E-04	2.57E-07	2.16E-07
	Total		1.90E+00		1.87E-05		3.37E+00		8.28E-06
Medium pollution	Cu	1.72E-05	4.27E-04			3.05E-05	7.58E-04		
	Mn	2.20E-05	1.54E+00			3.90E-05	2.72E+00		
	Pb	1.80E-05	5.13E-03			3.20E-05	9.09E-03		
	V	0.00E+00	0.00E+00			0.00E+00	0.00E+00		
	Zn	2.11E-04	7.03E-04			3.74E-04	1.25E-03		
	As	1.75E-06	5.83E-03	6.01E-07	9.08E-06	3.11E-06	1.03E-02	2.67E-07	4.02E-06
	Co	2.07E-06	3.63E-01	7.10E-07	6.96E-06	3.67E-06	6.43E-01	3.15E-07	3.08E-06
	Cr(VI)	1.64E-05	1.64E-01	5.61E-06	2.81E-06	2.90E-05	2.90E-01	2.49E-06	1.24E-06
	Ni	1.39E-06	6.73E-05	4.75E-07	3.99E-07	2.46E-06	1.19E-04	2.11E-07	1.77E-07
	Total		2.08E+00		1.92E-05		3.68E+00		8.53E-06
Heavy pollution	Cu	1.77E-05	4.40E-04			3.14E-05	7.80E-04		
	Mn	2.57E-05	1.80E+00			4.55E-05	3.18E+00		
	Pb	1.85E-05	5.27E-03			3.29E-05	9.34E-03		
	V	0.00E+00	0.00E+00			0.00E+00	0.00E+00		
	Zn	1.80E-04	6.00E-04			3.19E-04	1.06E-03		
	As	1.85E-06	6.15E-03	6.35E-07	9.58E-06	3.28E-06	1.09E-02	2.81E-07	4.25E-06
	Co	3.20E-06	5.60E-01	1.10E-06	1.08E-05	5.67E-06	9.94E-01	4.86E-07	4.77E-06
	Cr(VI)	1.95E-05	1.95E-01	6.67E-06	3.33E-06	3.45E-05	3.45E-01	2.96E-06	1.48E-06
	Ni	2.98E-06	1.45E-04	1.02E-06	8.59E-07	5.29E-06	2.57E-04	4.53E-07	3.81E-07
	Total		2.56E+00		2.45E-05		4.55E+00		1.09E-05

Table S11. Health risk related to cancer and non-cancer trace elements for different pollution levels during the heating period in 2019.

Pollution levels in heating period	Elements	Adults				Children			
		$ADD_{non-cancer}$	HQ	$LADD_{cancer}$	CR	$ADD_{non-cancer}$	HQ	$LADD_{cancer}$	CR
Clean	Cu	1.08E-05	2.69E-04			1.92E-05	4.77E-04		
	Mn	7.62E-06	5.33E-01			1.35E-05	9.45E-01		
	Pb	8.23E-06	2.34E-03			1.46E-05	4.15E-03		

	V	0.00E+00	0.00E+00			0.00E+00	0.00E+00		
	Zn	8.57E-05	2.86E-04			1.52E-04	5.06E-04		
	As	3.76E-07	1.25E-03	1.29E-07	1.95E-06	6.67E-07	2.22E-03	5.72E-08	8.63E-07
	Co	3.53E-06	6.19E-01	1.21E-06	1.19E-05	6.27E-06	1.10E+00	5.37E-07	5.26E-06
	Cr(VI)	1.14E-05	1.14E-01	3.91E-06	1.96E-06	2.02E-05	2.02E-01	1.74E-06	8.68E-07
	Ni	1.73E-06	8.40E-05	5.93E-07	4.98E-07	3.07E-06	1.49E-04	2.63E-07	2.21E-07
	Total		1.27E+00		1.63E-05		2.25E+00		7.22E-06
Light pollution	Cu	1.37E-05	3.40E-04			2.42E-05	6.03E-04		
	Mn	1.30E-05	9.09E-01			2.31E-05	1.61E+00		
	Pb	1.76E-05	5.01E-03			3.13E-05	8.88E-03		
	V	0.00E+00	0.00E+00			0.00E+00	0.00E+00		
	Zn	1.91E-04	6.38E-04			3.39E-04	1.13E-03		
	As	9.28E-07	3.08E-03	3.18E-07	4.80E-06	1.65E-06	5.47E-03	1.41E-07	2.13E-06
	Co	3.47E-06	6.08E-01	1.19E-06	1.17E-05	6.15E-06	1.08E+00	5.27E-07	5.17E-06
	Cr(VI)	2.39E-05	2.39E-01	8.20E-06	4.10E-06	4.24E-05	4.24E-01	3.63E-06	1.82E-06
	Ni	1.81E-06	8.78E-05	6.20E-07	5.21E-07	3.21E-06	1.56E-04	2.75E-07	2.31E-07
	Total		1.77E+00		2.11E-05		3.13E+00		9.35E-06
Medium pollution	Cu	1.62E-05	4.02E-04			2.87E-05	7.13E-04		
	Mn	2.06E-05	1.44E+00			3.65E-05	2.55E+00		
	Pb	2.23E-05	6.34E-03			3.96E-05	1.12E-02		
	V	0.00E+00	0.00E+00			0.00E+00	0.00E+00		
	Zn	3.06E-04	1.02E-03			5.43E-04	1.81E-03		
	As	1.76E-06	5.84E-03	6.02E-07	9.09E-06	3.12E-06	1.03E-02	2.67E-07	4.03E-06
	Co	3.09E-06	5.42E-01	1.06E-06	1.04E-05	5.48E-06	9.60E-01	4.70E-07	4.61E-06
	Cr(VI)	2.97E-05	2.97E-01	1.02E-05	5.09E-06	5.27E-05	5.27E-01	4.51E-06	2.26E-06
	Ni	1.76E-06	8.52E-05	6.02E-07	5.06E-07	3.11E-06	1.51E-04	2.67E-07	2.24E-07
	Total		2.29E+00		2.51E-05		4.07E+00		1.11E-05
Heavy pollution	Cu	2.31E-05	5.75E-04			4.10E-05	1.02E-03		
	Mn	2.20E-05	1.54E+00			3.91E-05	2.73E+00		
	Pb	2.01E-05	5.70E-03			3.56E-05	1.01E-02		
	V	0.00E+00	0.00E+00			0.00E+00	0.00E+00		
	Zn	2.16E-04	7.19E-04			3.83E-04	1.28E-03		
	As	1.27E-06	4.21E-03	4.34E-07	6.56E-06	2.25E-06	7.47E-03	1.93E-07	2.91E-06
	Co	3.28E-06	5.75E-01	1.13E-06	1.10E-05	5.83E-06	1.02E+00	4.99E-07	4.89E-06
	Cr(VI)	2.66E-05	2.66E-01	9.13E-06	4.56E-06	4.72E-05	4.72E-01	4.05E-06	2.02E-06
	Ni	2.69E-06	1.30E-04	9.21E-07	7.74E-07	4.76E-06	2.31E-04	4.08E-07	3.43E-07
	Total		2.39E+00		2.29E-05		4.24E+00		1.02E-05

Table S12. Health risk related to cancer and non-cancer trace elements for different pollution levels before the heating period in 2020.

Elements	Adults	Children

Pollution levels before heating period		$ADD_{non\text{-}cancer}$	HQ	$LADD_{cancer}$	CR	$ADD_{non\text{-}cancer}$	HQ	$LADD_{cancer}$	CR
Clean	Cu	1.79E-06	4.45E-05			3.17E-06	7.89E-05		
	Mn	8.89E-06	6.22E-01			1.58E-05	1.10E+00		
	Pb	5.06E-06	1.44E-03			8.97E-06	2.55E-03		
	V	4.17E-08	5.96E-06			7.40E-08	1.06E-05		
	Zn	2.15E-05	7.16E-05			3.81E-05	1.27E-04		
	As	1.59E-06	5.27E-03	5.44E-07	8.22E-06	2.82E-06	9.35E-03	2.41E-07	3.64E-06
	Co	6.72E-08	1.18E-02	2.30E-08	2.26E-07	1.19E-07	2.09E-02	1.02E-08	1.00E-07
	Cr(VI)	9.59E-06	9.59E-02	3.29E-06	1.64E-06	1.70E-05	1.70E-01	1.46E-06	7.29E-07
	Ni	6.82E-07	3.31E-05	2.34E-07	1.96E-07	1.21E-06	5.87E-05	1.04E-07	8.70E-08
	Total		7.37E-01		1.03E-05		1.31E+00		4.57E-06
Light pollution	Cu	1.08E-05	2.68E-04			1.91E-05	4.76E-04		
	Mn	1.94E-05	1.35E+00			3.44E-05	2.40E+00		
	Pb	1.29E-05	3.67E-03			2.29E-05	6.52E-03		
	V	0.00E+00	0.00E+00			0.00E+00	0.00E+00		
	Zn	4.72E-05	1.57E-04			8.37E-05	2.79E-04		
	As	2.37E-06	7.89E-03	8.14E-07	1.23E-05	4.21E-06	1.40E-02	3.61E-07	5.45E-06
	Co	4.40E-07	7.71E-02	1.51E-07	1.48E-06	7.80E-07	1.37E-01	6.69E-08	6.56E-07
	Cr(VI)	1.79E-05	1.79E-01	6.14E-06	3.07E-06	3.18E-05	3.18E-01	2.72E-06	1.36E-06
	Ni	1.89E-06	9.20E-05	6.49E-07	5.46E-07	3.36E-06	1.63E-04	2.88E-07	2.42E-07
	Total		1.62E+00		1.74E-05		2.88E+00		7.71E-06
Medium pollution	Cu	1.45E-05	3.60E-04			2.57E-05	6.38E-04		
	Mn	2.31E-05	1.62E+00			4.10E-05	2.87E+00		
	Pb	1.46E-05	4.13E-03			2.58E-05	7.33E-03		
	V	0.00E+00	0.00E+00			0.00E+00	0.00E+00		
	Zn	5.96E-05	1.99E-04			1.06E-04	3.52E-04		
	As	2.24E-06	7.45E-03	7.69E-07	1.16E-05	3.98E-06	1.32E-02	3.41E-07	5.15E-06
	Co	7.44E-07	1.30E-01	2.55E-07	2.50E-06	1.32E-06	2.31E-01	1.13E-07	1.11E-06
	Cr(VI)	2.21E-05	2.21E-01	7.58E-06	3.79E-06	3.92E-05	3.92E-01	3.36E-06	1.68E-06
	Ni	2.10E-06	1.02E-04	7.19E-07	6.04E-07	3.72E-06	1.80E-04	3.19E-07	2.68E-07
	Total		1.98E+00		1.85E-05		3.51E+00		8.21E-06
Heavy pollution	Cu	8.59E-06	2.14E-04			1.52E-05	3.79E-04		
	Mn	2.12E-05	1.48E+00			3.75E-05	2.62E+00		
	Pb	1.20E-05	3.41E-03			2.13E-05	6.05E-03		
	V	0.00E+00	0.00E+00			0.00E+00	0.00E+00		
	Zn	5.64E-05	1.88E-04			1.00E-04	3.33E-04		
	As	2.60E-06	8.65E-03	8.92E-07	1.35E-05	4.61E-06	1.53E-02	3.96E-07	5.97E-06

	Co	1.86E-07	3.25E-02	6.37E-08	6.24E-07	3.29E-07	5.77E-02	2.82E-08	2.77E-07
	Cr(VI)	2.38E-05	2.38E-01	8.15E-06	4.07E-06	4.21E-05	4.21E-01	3.61E-06	1.81E-06
	Ni	1.71E-06	8.29E-05	5.86E-07	4.92E-07	3.03E-06	1.47E-04	2.60E-07	2.18E-07
	Total		1.76E+00		1.87E-05		3.12E+00		8.28E-06

Table S13. Health risk related to cancer and non-cancer trace elements for different pollution levels during the heating period in 2020.

Pollution levels in heating period	Elements	Adults				Children			
		$ADD_{non-cancer}$	HQ	$LADD_{cancer}$	CR	$ADD_{non-cancer}$	HQ	$LADD_{cancer}$	CR
Clean	Cu	3.62E-05	9.01E-04			6.42E-05	1.60E-03		
	Mn	1.58E-05	1.10E+00			2.79E-05	1.95E+00		
	Pb	1.02E-05	2.90E-03			1.81E-05	5.15E-03		
	V	2.50E-07	3.57E-05			4.44E-07	6.34E-05		
	Zn	4.99E-05	1.66E-04			8.84E-05	2.95E-04		
	As	6.73E-07	2.24E-03	2.31E-07	3.48E-06	1.19E-06	3.96E-03	1.02E-07	1.54E-06
	Co	8.93E-07	1.56E-01	3.06E-07	3.00E-06	1.58E-06	2.77E-01	1.36E-07	1.33E-06
	Cr(VI)	1.12E-05	1.12E-01	3.84E-06	1.92E-06	1.99E-05	1.99E-01	1.70E-06	8.52E-07
	Ni	2.02E-06	9.79E-05	6.91E-07	5.81E-07	3.58E-06	1.74E-04	3.07E-07	2.57E-07
	Total		1.38E+00		8.99E-06		2.44E+00		3.98E-06
Light pollution	Cu	4.97E-05	1.24E-03			8.82E-05	2.19E-03		
	Mn	2.35E-05	1.64E+00			4.16E-05	2.91E+00		
	Pb	1.73E-05	4.91E-03			3.06E-05	8.70E-03		
	V	0.00E+00	0.00E+00			0.00E+00	0.00E+00		
	Zn	8.15E-05	2.72E-04			1.45E-04	4.82E-04		
	As	8.15E-07	2.71E-03	2.79E-07	4.22E-06	1.45E-06	4.80E-03	1.24E-07	1.87E-06
	Co	6.43E-07	1.13E-01	2.21E-07	2.16E-06	1.14E-06	2.00E-01	9.78E-08	9.58E-07
	Cr(VI)	1.82E-05	1.82E-01	6.23E-06	3.11E-06	3.22E-05	3.22E-01	2.76E-06	1.38E-06
	Ni	1.47E-06	7.15E-05	5.05E-07	4.24E-07	2.61E-06	1.27E-04	2.24E-07	1.88E-07
	Total		1.94E+00		9.92E-06		3.45E+00		4.40E-06
Medium pollution	Cu	4.55E-05	1.13E-03			8.06E-05	2.01E-03		
	Mn	2.50E-05	1.75E+00			4.43E-05	3.10E+00		
	Pb	1.53E-05	4.34E-03			2.71E-05	7.70E-03		
	V	0.00E+00	0.00E+00			0.00E+00	0.00E+00		
	Zn	7.52E-05	2.51E-04			1.33E-04	4.44E-04		
	As	9.82E-07	3.26E-03	3.37E-07	5.09E-06	1.74E-06	5.79E-03	1.49E-07	2.25E-06
	Co	5.54E-07	9.70E-02	1.90E-07	1.86E-06	9.82E-07	1.72E-01	8.42E-08	8.25E-07
	Cr(VI)	1.93E-05	1.93E-01	6.60E-06	3.30E-06	3.42E-05	3.42E-01	2.93E-06	1.46E-06
	Ni	2.09E-06	1.02E-04	7.18E-07	6.03E-07	3.71E-06	1.80E-04	3.18E-07	2.67E-07
	Total		2.05E+00		1.09E-05		3.63E+00		4.81E-06
Heavy pollution	Cu	3.74E-05	9.30E-04			6.63E-05	1.65E-03		

	Mn	2.00E-05	1.40E+00			3.54E-05	2.48E+00		
	Pb	9.90E-06	2.81E-03			1.76E-05	4.99E-03		
	V	0.00E+00	0.00E+00			0.00E+00	0.00E+00		
	Zn	4.09E-05	1.36E-04			7.26E-05	2.42E-04		
	As	4.92E-07	1.64E-03	1.69E-07	2.55E-06	8.73E-07	2.90E-03	7.48E-08	1.13E-06
	Co	1.12E-06	1.96E-01	3.83E-07	3.76E-06	1.98E-06	3.47E-01	1.70E-07	1.67E-06
	Cr(VI)	1.24E-05	1.24E-01	4.25E-06	2.12E-06	2.20E-05	2.20E-01	1.88E-06	9.41E-07
	Ni	2.78E-06	1.35E-04	9.55E-07	8.02E-07	4.94E-06	2.40E-04	4.23E-07	3.56E-07
	Total		1.72E+00		9.23E-06		3.06E+00		4.09E-06

Table S14. Health risk related to cancer and non-cancer trace elements for different pollution levels before the heating period in 2021.

Pollution levels before heating period	Elements	Adults				Children			
		$ADD_{non\text{-}cancer}$	HQ	$LADD_{cancer}$	CR	$ADD_{non\text{-}cancer}$	HQ	$LADD_{cancer}$	CR
Clean	Cu	9.89E-06	2.46E-04			1.75E-05	4.36E-04		
	Mn	8.56E-06	5.98E-01			1.52E-05	1.06E+00		
	Pb	5.64E-06	1.60E-03			9.99E-06	2.84E-03		
	V	3.91E-07	5.58E-05			6.93E-07	9.90E-05		
	Zn	2.49E-05	8.31E-05			4.42E-05	1.47E-04		
	As	8.59E-07	2.85E-03	2.95E-07	4.45E-06	1.52E-06	5.06E-03	1.31E-07	1.97E-06
	Co	1.56E-06	2.74E-01	5.36E-07	5.25E-06	2.77E-06	4.85E-01	2.38E-07	2.33E-06
	Cr(VI)	5.70E-06	5.70E-02	1.95E-06	9.77E-07	1.01E-05	1.01E-01	8.66E-07	4.33E-07
	Ni	1.62E-06	7.86E-05	5.55E-07	4.66E-07	2.87E-06	1.39E-04	2.46E-07	2.07E-07
	Total		9.35E-01		1.28E-05		1.66E+00		5.69E-06
Light pollution	Cu	1.20E-05	2.99E-04			2.13E-05	5.30E-04		
	Mn	1.87E-05	1.31E+00			3.32E-05	2.32E+00		
	Pb	1.14E-05	3.24E-03			2.03E-05	5.75E-03		
	V	0.00E+00	0.00E+00			0.00E+00	0.00E+00		
	Zn	4.92E-05	1.64E-04			8.72E-05	2.91E-04		
	As	1.26E-06	4.20E-03	4.34E-07	6.55E-06	2.24E-06	7.45E-03	1.92E-07	7.45E-03
	Co	2.56E-06	4.48E-01	8.77E-07	8.59E-06	4.53E-06	7.94E-01	3.89E-07	3.81E-06
	Cr(VI)	1.29E-05	1.29E-01	4.41E-06	2.20E-06	2.28E-05	2.28E-01	1.95E-06	9.77E-07
	Ni	1.70E-06	8.25E-05	5.82E-07	4.89E-07	3.01E-06	1.46E-04	2.58E-07	2.17E-07
	Total		1.90E+00		1.90E-05		3.36E+00		8.41E-06
Medium pollution	Cu	2.18E-06	5.41E-05			3.86E-06	9.59E-05		
	Mn	1.94E-05	1.36E+00			3.44E-05	2.41E+00		
	Pb	1.16E-05	3.29E-03			2.06E-05	5.84E-03		
	V	0.00E+00	0.00E+00			0.00E+00	0.00E+00		

	Zn	5.71E-05	1.90E-04			1.01E-04	3.38E-04		
	As	1.05E-06	3.49E-03	3.60E-07	5.43E-06	1.86E-06	6.18E-03	1.60E-07	2.41E-06
	Co	1.74E-06	3.05E-01	5.97E-07	5.85E-06	3.09E-06	5.41E-01	2.65E-07	2.59E-06
	Cr(VI)	1.46E-05	1.46E-01	5.00E-06	2.50E-06	2.59E-05	2.59E-01	2.22E-06	1.11E-06
	Ni	1.98E-06	9.61E-05	6.79E-07	5.70E-07	3.51E-06	1.70E-04	3.01E-07	2.53E-07
	Total		1.82E+00		1.44E-05		3.22E+00		6.37E-06
Heavy pollution	Cu	8.64E-07	2.15E-05			1.53E-06	3.81E-05		
	Mn	1.73E-05	1.21E+00			3.06E-05	2.14E+00		
	Pb	1.30E-05	3.69E-03			2.30E-05	6.54E-03		
	V	0.00E+00	0.00E+00			0.00E+00	0.00E+00		
	Zn	5.76E-05	1.92E-04			1.02E-04	3.40E-04		
	As	1.15E-06	3.82E-03	3.94E-07	5.95E-06	2.04E-06	6.77E-03	1.75E-07	2.64E-06
	Co	2.66E-06	4.66E-01	9.13E-07	8.95E-06	4.72E-06	8.27E-01	4.05E-07	3.97E-06
	Cr(VI)	1.57E-05	1.57E-01	5.38E-06	2.69E-06	2.78E-05	2.78E-01	2.39E-06	1.19E-06
	Ni	1.10E-06	5.32E-05	3.76E-07	3.16E-07	1.94E-06	9.44E-05	1.67E-07	1.40E-07
	Total		1.84E+00		1.79E-05		3.26E+00		7.94E-06

Table S15. Health risk related to cancer and non-cancer trace elements for different pollution levels during the heating period in 2021.

Pollution levels in heating period	Elements	Adults				Children			
		$ADD_{non-cancer}$	HQ	$LADD_{cancer}$	CR	$ADD_{non-cancer}$	HQ	$LADD_{cancer}$	CR
Clean	Cu	6.36E-06	1.58E-04			1.13E-05	2.81E-04		
	Mn	1.06E-05	7.43E-01			1.88E-05	1.32E+00		
	Pb	7.38E-06	2.10E-03			1.31E-05	3.72E-03		
	V	3.29E-07	4.71E-05			5.84E-07	8.35E-05		
	Zn	4.34E-05	1.45E-04			7.70E-05	2.57E-04		
	As	1.27E-06	4.22E-03	4.36E-07	6.58E-06	2.25E-06	7.49E-03	1.93E-07	2.92E-06
	Co	8.45E-07	1.48E-01	2.90E-07	2.84E-06	1.50E-06	2.62E-01	1.28E-07	1.26E-06
	Cr(VI)	8.67E-06	8.67E-02	2.97E-06	1.49E-06	1.54E-05	1.54E-01	1.32E-06	6.59E-07
	Ni	2.83E-06	1.37E-04	9.70E-07	8.15E-07	5.02E-06	2.44E-04	4.30E-07	3.61E-07
	Total		9.85E-01		1.17E-05		1.75E+00		5.20E-06
Light pollution	Cu	8.68E-06	2.16E-04			1.54E-05	3.83E-04		
	Mn	2.23E-05	1.56E+00			3.96E-05	2.77E+00		
	Pb	1.33E-05	3.79E-03			2.36E-05	6.72E-03		
	V	0.00E+00	0.00E+00			0.00E+00	0.00E+00		
	Zn	8.54E-05	2.85E-04			1.51E-04	5.05E-04		
	As	2.33E-06	7.75E-03	8.00E-07	1.21E-05	4.14E-06	1.37E-02	3.55E-07	5.36E-06

	Co	7.37E-07	1.29E-01	2.53E-07	2.48E-06	1.31E-06	2.29E-01	1.12E-07	1.10E-06
	Cr(VI)	1.84E-05	1.84E-01	6.31E-06	3.16E-06	3.26E-05	3.26E-01	2.80E-06	1.40E-06
	Ni	3.79E-06	1.84E-04	1.30E-06	1.09E-06	6.71E-06	3.26E-04	5.75E-07	4.83E-07
	Total		1.89E+00		1.88E-05		3.35E+00		8.34E-06
Medium pollution	Cu	5.50E-06	1.37E-04			9.76E-06	2.43E-04		
	Mn	2.55E-05	1.79E+00			4.53E-05	3.17E+00		
	Pb	1.75E-05	4.96E-03			3.10E-05	8.80E-03		
	V	0.00E+00	0.00E+00			0.00E+00	0.00E+00		
	Zn	9.09E-05	3.03E-04			1.61E-04	5.37E-04		
	As	2.57E-06	8.54E-03	8.82E-07	1.33E-05	4.56E-06	1.51E-02	3.91E-07	5.90E-06
	Co	6.60E-07	1.16E-01	2.26E-07	2.22E-06	1.17E-06	2.05E-01	1.00E-07	9.84E-07
	Cr(VI)	2.09E-05	2.09E-01	7.16E-06	3.58E-06	3.70E-05	3.70E-01	3.18E-06	1.59E-06
	Ni	3.66E-06	1.78E-04	1.25E-06	1.05E-06	6.49E-06	3.15E-04	5.56E-07	4.67E-07
	Total		2.12E+00		2.02E-05		3.77E+00		8.94E-06
Heavy pollution	Cu	7.04E-06	1.75E-04			1.25E-05	3.10E-04		
	Mn	2.64E-05	1.85E+00			4.69E-05	3.28E+00		
	Pb	1.79E-05	5.08E-03			3.17E-05	9.01E-03		
	V	0.00E+00	0.00E+00			0.00E+00	0.00E+00		
	Zn	9.27E-05	3.09E-04			1.64E-04	5.48E-04		
	As	2.21E-06	7.33E-03	7.56E-07	1.14E-05	3.91E-06	1.30E-02	3.35E-07	5.06E-06
	Co	8.24E-07	1.44E-01	2.83E-07	2.77E-06	1.46E-06	2.56E-01	1.25E-07	1.23E-06
	Cr(VI)	2.18E-05	2.18E-01	7.48E-06	3.74E-06	3.87E-05	3.87E-01	3.32E-06	1.66E-06
	Ni	3.45E-06	1.68E-04	1.18E-06	9.94E-07	6.12E-06	2.97E-04	5.25E-07	4.41E-07
	Total		2.22E+00		1.89E-05		3.94E+00		8.39E-06

1. US EPA. Risk Assessment Guidance for Superfund. In: Part A: Human Health Evaluation Manual; Part E, Supplemental Guidance for Dermal Risk Assessment; Part F, Supplemental Guidance for Inhalation Risk Assessment, vol. I, **2011**.
2. Feng, J.L.; Yu, H.; Su, X.F.; Liu, S.H.; Li, Y.; Pan, Y.P.; Sun, J.H. Chemical composition and source apportionment of $PM_{2.5}$ during Chinese Spring Festival at Xinxiang, a heavily polluted city in North China: Fireworks and health risks. *Atmos. Res*. **2016**, 182, 176–188.
3. Alves, R.; Machado, G.; Zagui, G.; Bandeira, O.; Santos, D.; Nadal, M.; Sierra, J.;Domingo, J.; Segura-Mu noz, S. Metals risk assessment for children's health in water and particulate matter in a southeastern Brazilian city. *Environ. Res.* **2019**, 177, 108623.
4. Chen, H.; Lu, X.W.; Li, L.Y. Spatial distribution and risk assessment of metals in dust based on samples from nursery and primary schools of Xi'an, China. *Atmos. Environ.* **2014**, 88, 172-182.
5. Wang, B.; Su, Y.; Tian, L.Y.; Peng, S.C.; Ji, R. Heavy metals in face paints: assessment of the health risks to Chinese opera actors. *Sci. Total Environ.* **2020**, 724, 138163.
